# MED12 Dictates Epithelial Ovarian Cancer Cell Ferroptosis Sensitivity via YAP–TEAD1 Signaling

**DOI:** 10.3390/ijms27021020

**Published:** 2026-01-20

**Authors:** Xiaolin Luo, Yi Ding, Zeying Wang, Jihong Liu

**Affiliations:** Department of Gynecologic Oncology, State Key Laboratory of Oncology in South China, Guangdong Provincial Clinical Research Center for Cancer, Sun Yat-Sen University Cancer Center, Guangzhou 510060, China; luoxl@sysucc.org.cn (X.L.); dingyi1@sysucc.org.cn (Y.D.); wangzy3@sysucc.org.cn (Z.W.)

**Keywords:** MED12, ferroptosis, YAP–TEAD1 signaling, ovarian cancer, chemotherapy resistance

## Abstract

Epithelial ovarian cancer (EOC) represents the most lethal malignancy arising from the female reproductive tract, largely due to the clinical challenge of chemotherapy resistance. Recent studies indicate that ferroptosis—a distinct form of programmed cell death driven by iron accumulation and lipid peroxidation, could potentially exploit a vulnerability in chemoresistant cancer cells. Here, we identify MED12 as a critical regulator of ferroptosis sensitivity in EOC through modulation of the YAP–TEAD1 signaling pathway. Using CRISPR/Cas9-mediated knockout and rescue experiments in EOC cell lines, we demonstrate that MED12 deficiency significantly enhances sensitivity to ferroptosis inducers (RSL3 and Erastin), as evidenced by reduced IC50 values. Transcriptomic and chromatin accessibility analyses reveal that MED12 loss activates YAP signaling through TEAD1 upregulation, increasing chromatin accessibility at YAP–TEAD1 target loci and elevating the expression of downstream effectors CYR61 and CTGF. Pharmacological inhibition of YAP with verteporfin or siRNA-mediated TEAD1 knockdown reverses ferroptosis sensitivity in MED12-deficient cells, confirming pathway specificity. These findings establish MED12 as a modulator of the YAP–TEAD1–ferroptosis axis and suggest that targeting this pathway could overcome chemoresistance in MED12-deficient EOC. Our work provides a mechanistic foundation for exploiting ferroptosis induction as a therapeutic strategy in ovarian cancer.

## 1. Introduction

Epithelial ovarian cancer (EOC), which represents about 90% of ovarian malignancies, has an annual global incidence of 6.6 per 100,000 women. Its mortality rate is notably high at 4.2 per 100,000, underscoring the disease’s aggressive nature and the common challenge of late detection [1,2,3]. Although most patients respond initially to platinum-based chemotherapy, 70% relapse within two years. And, upon recurrence, the majority will acquire platinum-resistant disease [1,4,5]. Chemoresistance in EOC arises from multifactorial mechanisms, including altered drug efflux, DNA repair dysregulation, tumor microenvironment, and so on [1,4,5]. Until now, the precise way in which EOC cells develop chemoresistance has remained unclear, and methods of eliminating chemoresistant EOC cells are still lacking.

Characterized specifically by the fatal buildup of peroxidized lipids, ferroptosis represents a distinct mode of programmed cell death that is strictly dependent on iron [6,7]. This process is mechanistically distinct from apoptosis and necroptosis, involving the accumulation of reactive oxygen species through Fenton reactions and the oxidation of polyunsaturated fatty acids in cellular membranes [6,8,9,10]. Emerging evidence indicates that chemotherapy-resistant cancer cells frequently exhibit increased vulnerability to ferroptosis induction [8,9]. It has been reported that chemoresistant subclones may enrich polyunsaturated fatty acid phospholipids through ACSL4 upregulation, creating abundant peroxidation substrates [11].

Mediator complex subunit 12 (MED12), an evolutionarily conserved component of the Mediator complex, functions as a key regulator within the RNA polymerase II transcriptional apparatus [12,13]. Recent studies have implicated MED12 as a pivotal modulator of chemotherapy resistance through multifaceted mechanisms [14,15,16,17,18,19]. MED12 dysfunction (e.g., mutations or downregulation) frequently correlates with enhanced survival pathways. Loss of MED12 activates TGF-β/SMAD signaling, promoting epithelial–mesenchymal transition (EMT) and upregulating ATP-binding cassette (ABC) transporters such as ABCB1, thereby facilitating drug efflux [12,18]. Our published research also suggested that the loss of MED12 induces chemoresistance and dormancy in EOC cells [19]. While MED12 somatic mutations are relatively infrequent in EOC, TCGA data suggested that the expression of MED12 was lower in ovarian cancers than normal samples and low MED12 expression is associated with poorer disease-free survival. However, whether MED12 can regulate ferroptosis sensitivity of EOC cells is still unknown.

In this study, we elucidated the relationship between MED12 and ferroptosis susceptibility in EOC. We demonstrated that MED12 knockout significantly enhances ferroptosis sensitivity in ovarian cancer cells. Mechanistically, MED12 depletion activates the YAP signaling pathway to modulate ferroptosis response. Further molecular analysis reveals that MED12 regulates the expression of TEAD1, the core transcriptional factor of the YAP signaling pathway, thereby promoting chromatin accessibility at YAP target gene loci. These findings will deepen our understanding of the regulatory mechanisms governing ferroptosis sensitivity in ovarian cancer cells, and provide a theoretical foundation for developing novel therapeutic strategies to eliminate chemotherapy-resistant EOC cells through ferroptosis induction.

## 2. Results

### 2.1. Enhanced Ferroptosis Sensitivity in MED12-Knockout Chemoresistant Epithelial Ovarian Cancer Cells

**Figure 1:**
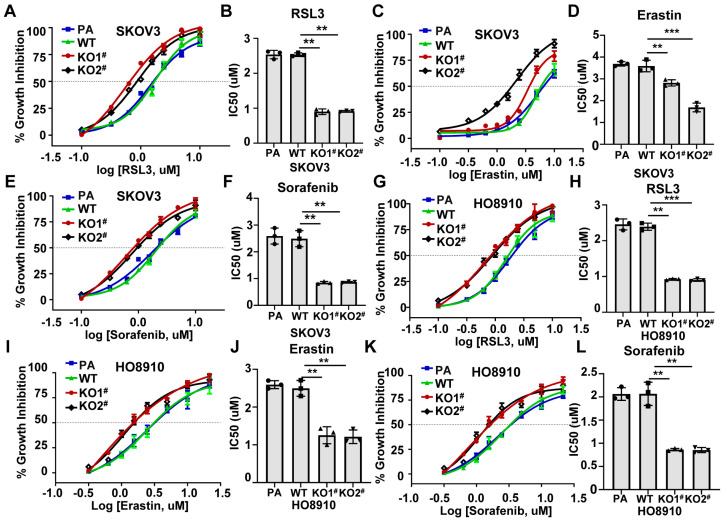
MED12 knockout enhances ferroptosis sensitivity in ovarian cancer cells. (**A**,**B**) Dose–response curves and IC50 for RSL3 treatment in WT and MED12-KO SKOV3 cells. ** *p* < 0.01. (**C**,**D**) Dose–response curves and IC50 for Erastin treatment in WT and MED12-KO SKOV3 cells. ** *p* < 0.01 and *** *p* < 0.001. (**E**,**F**) Dose–response curves and IC50 for Sorafenib treatment in WT and MED12-KO SKOV3 cells. ** *p* < 0.01. (**G**,**H**) Dose–response curves and IC50 and representative cell images for RSL3 treatment in WT and MED12-KO HO8910 cells. ** *p* < 0.01 and *** *p* < 0.001. (**I**,**J**) Dose–response curves and IC50 and representative cell images for Erastin treatment in WT and MED12-KO HO8910 cells. ** *p* < 0.01. (**K**,**L**) Dose–response curves and IC50 for Sorafenib treatment in WT and MED12-KO HO8910 cells. ** *p* < 0.01. One-way ANOVA test was used to determine statistical significance. PA: parental cells; WT: wild-type; KO: knockout. Error bars represent SD (*n* = 3).

Mounting evidence indicates that chemotherapy-resistant tumor cells develop heightened susceptibility to ferroptosis inducers due to metabolic vulnerabilities arising from drug resistance adaptations [8,9]. Building upon this paradigm, we wanted to investigate whether MED12 knockout, which confers chemoresistance in ovarian cancer cells (Figure S1), similarly enhances ferroptosis sensitivity. Using two well-characterized epithelial ovarian cancer cell lines (SKOV3 and HO8910) with CRISPR/Cas9-mediated MED12 knockout (KO) (Figure S2), we treated cells with the canonical ferroptosis inducers RSL3 (a GPX4 inhibitor), Erastin (a system xc-blocker), and Sorafenib across an eight-dose concentration range (0.1–10 μM). Dose–response curves generated over 48 h revealed striking differences in ferroptosis sensitivity: MED12-KO cells exhibited significantly lower IC50 values for both compounds versus wild-type (WT) controls: SKOV3 MED12-KO: RSL3 IC50 = 0.8 ± 0.21 μM or 0.86 ± 0.24 μM (vs. WT 2.6 ± 0.74 μM, *p* < 0.01); Erastin: IC50 = 6.1 ± 0.73 μM or 2.7 ± 0.16 μM (vs. WT 7.7 ± 0.97 μM, *p* < 0.01 or *p* < 0.001); Sorafenib: IC50 = 0.81 ± 0.23 μM or 0.87 ± 0.26 μM (vs. WT 2.7 ± 0.37 μM, *p* < 0.01) (Figure 1A–F and Table S1). HO8910 MED12-KO: RSL3 IC50 = 0.93 ± 0.22 μM or 0.92 ± 0.12 μM (vs. WT 2.6 ± 0.3 μM, *p* < 0.01); Erastin: IC50 = 2.7 ± 0.33 μM or 2.4 ± 0.36 μM (vs. WT 5.2 ± 0.67 μM, *p* < 0.01); Sorafenib: IC50 = 2.8 ± 0.43 μM or 2.82 ± 0.46 μM (vs. WT 5.7 ± 0.67 μM, *p* < 0.01) (Figure 1G–L and Table S1). These results indicate that MED12-knockout will enhance the ferroptosis sensitivity of EOC cells.

Next, we performed MED12 overexpression experiments in HO8910, SKOV3, and normal epithelial ovarian cell IOSE80. However, we found that MED12 overexpression nearly does not change the sensitivity of these cells to ferroptosis-inducing agents (Figure S3). The reason may be that MED12 is part of the Mediator complex, and the overexpression of MED12 alone did not change the function of the Mediator complex.

### 2.2. Rescue of MED12 Expression Reverses Ferroptosis Sensitivity in Ovarian Cancer Cells

**Figure 2:**
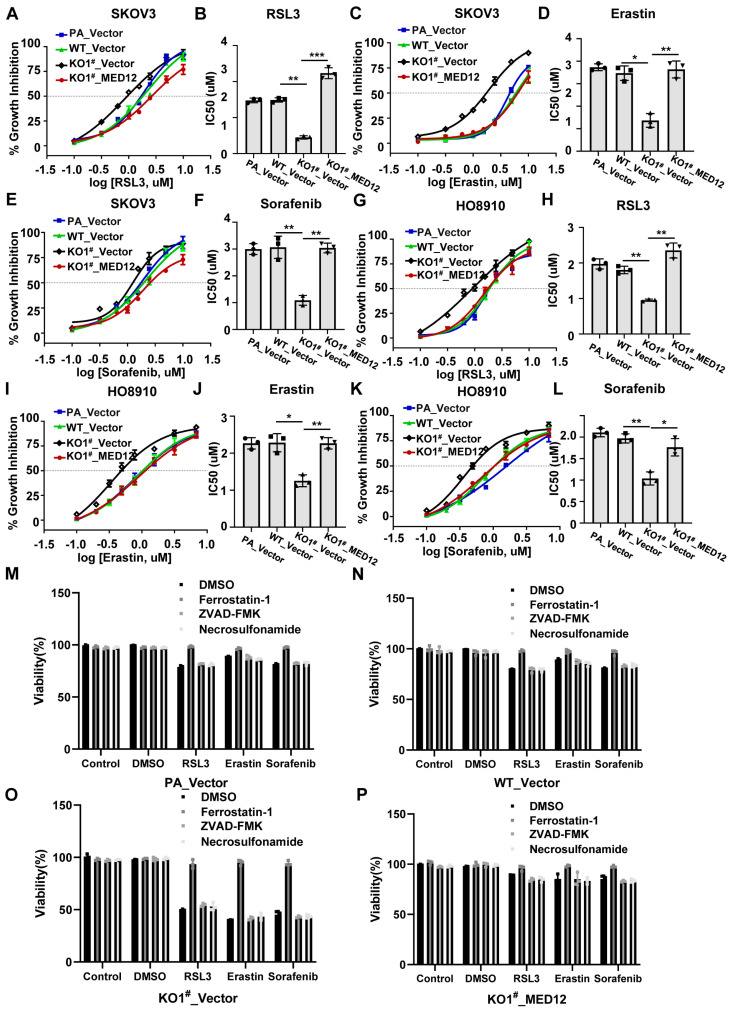
MED12 re-expression rescues ferroptosis sensitivity. (**A**,**B**) Dose–response curves and IC50 for RSL3 in SKOV3 MED12-reconstituted cells versus controls. ** *p* < 0.01 and *** *p* < 0.001. (**C**,**D**) Dose–response curves and IC50 for Erastin in SKOV3 MED12-reconstituted cells versus controls. * *p* < 0.05 and ** *p* < 0.01. (**E**,**F**) Dose–response curves and IC50 for Sorafenib in SKOV3 MED12-reconstituted cells versus controls. ** *p* < 0.01. (**G**,**H**) Dose–response curves and IC50 for RSL3 in HO8910 MED12-reconstituted cells versus controls. ** *p* < 0.01. (**I**,**J**) Dose–response curves and IC50 for Erastin in HO8910 MED12-reconstituted cells versus controls. * *p* < 0.05 and ** *p* < 0.01. (**K**,**L**) Dose–response curves and IC50 for Sorafenib in HO8910 MED12-reconstituted cells versus controls. * *p* < 0.05 and ** *p* < 0.01. (**M**–**P**) Cell death inhibition assays showing ferroptosis specificity in SKOV3. One-way ANOVA test was used to determine statistical significance. PA: parental cells; WT: wild-type; KO: knockout. Error bars represent SD (*n* = 3).

To definitively establish the functional role of MED12 in regulating ferroptosis susceptibility, we performed genetic rescue experiments in MED12-knockout (MED12-KO) SKOV3 and HO8910 cells. Lentiviral-mediated re-expression of wild-type MED12 (confirmed by Western blot in Figure S4A,B) significantly attenuated the enhanced ferroptosis sensitivity observed in MED12-KO cells. MED12-reconstituted cells exhibited IC50 values comparable to wild-type (WT) controls (Figure 2A–L and Table S1). Critically, we conducted comprehensive inhibitor profiling to unequivocally establish the ferroptotic nature of the observed cell death. The cell death phenotype was confirmed as ferroptosis, as it was selectively suppressed by Ferrostatin-1; however, this effect was not observed with Z-VAD-FMK (an apoptosis blocker) or Necrosulfonamide (which targets necroptosis) (Figure 2M–P and Figure S4C–F). Taken together, these findings suggest that MED12 can regulate ferroptosis sensitivity in ovarian cancer cells.

### 2.3. Transcriptomic Profiling Reveals Activation of the YAP Pathway in MED12-KO Ovarian Cancer Cells

**Figure 3:**
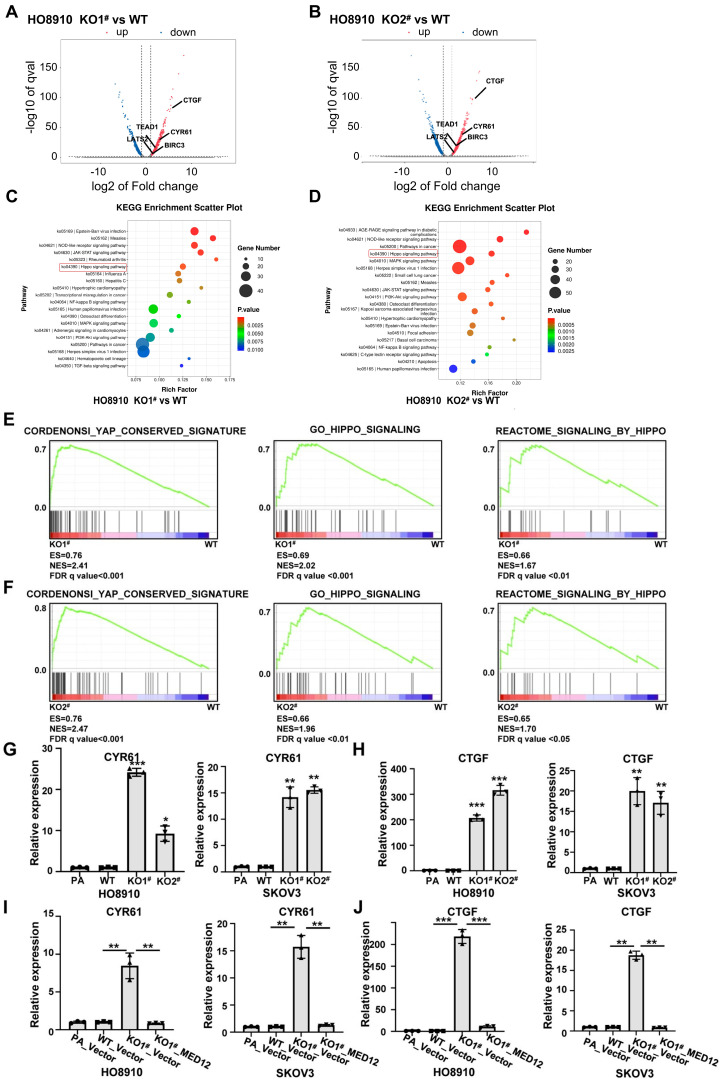
Transcriptomic profiling reveals MED12 regulation of Hippo–YAP signaling. (**A**,**B**) Volcano plot of differentially expressed genes (|log2FC| > 1, *q* value < 0.05) in MED12-KO versus WT HO8910 cells. (**C**,**D**) KEGG pathway enrichment analysis of upregulated genes in MED12-KO cells. (**E**,**F**) GSEA plot showing enrichment of Hippo–YAP signaling pathways in MED12-KO cells. (**G**,**H**) qRT-PCR assay of CYR61 and CTGF expression in MED12-KO SKOV3 and HO8910 cells. * *p* < 0.05, ** *p* < 0.01, and *** *p* < 0.001. (**I**,**J**) qRT-PCR assay of CYR61 and CTGF expression in MED12-KO and MED12-reconstituted SKOV3 and HO8910 cells. ** *p* < 0.01 and *** *p* < 0.001. One-way ANOVA test was used to determine statistical significance. PA: parental cells; WT: wild-type; KO: knockout. Error bars represent SD (*n* = 3).

To investigate the molecular mechanisms underlying MED12-mediated regulation of ferroptosis sensitivity in ovarian cancer cells, we performed comprehensive transcriptomic analysis using RNA sequencing (RNA-seq). Three isogenic HO8910 cell lines were compared: (1) wild-type control (WT); (2) MED12 knockout 1 (KO1); (3) MED12 knockout 2 (KO2) (Figure 3A,B). MED12 knockout significantly altered gene expression (Figure 3A,B and Table S2). Significantly upregulated genes in MED12-KO cells compared to WT cells included some Hippo–YAP pathway associated genes, such as CTGF, CYR61, and TEAD1 (Figure 3A,B). Bioinformatic analysis revealed significant pathway alterations in MED12-KO cells. The Hippo–YAP signaling pathway emerged as one of the most significantly enriched pathways in both MED12-KO cells compared to WT HO8910 cells (Figure 3C,D and Tables S3 and S4). Gene Set Enrichment Analysis (GSEA) confirmed significant activation of Hippo–YAP pathway in MED12-KO cells (Figure 3E,F). The top enriched gene sets included the following: YAP_CONSERVED_SIGNATURE (FDR *q* value < 0.001), GO_HIPPO_SIGNALING (FDR *q* value < 0.001 or 0.01), and REACTOME_SIGNALING_BY_HIPPO (FDR *q* value < 0.01 or 0.05). qRT-PCR and a Western blot assay validated that MED12 knockout increased the expression of Hippo–YAP pathway downstream target genes CYR61 and CTGF in HO8910 and SKOV3 cells (Figure 3G,H and Figure S5A,B). Importantly, lentiviral-mediated re-expression of wild-type MED12 significantly attenuated the increasing expression of CYR61 and CTGF in MED12-KO cells (Figure 3I,J and Figure S5C,D). Overall, the concordance between KEGG, GSEA, and qPCR data provides compelling evidence that MED12 serves as a master regulator of Hippo–YAP signaling in ovarian cancer.

### 2.4. MED12 Regulates Ferroptosis Sensitivity in Ovarian CANCER Cells via YAP Pathway

**Figure 4:**
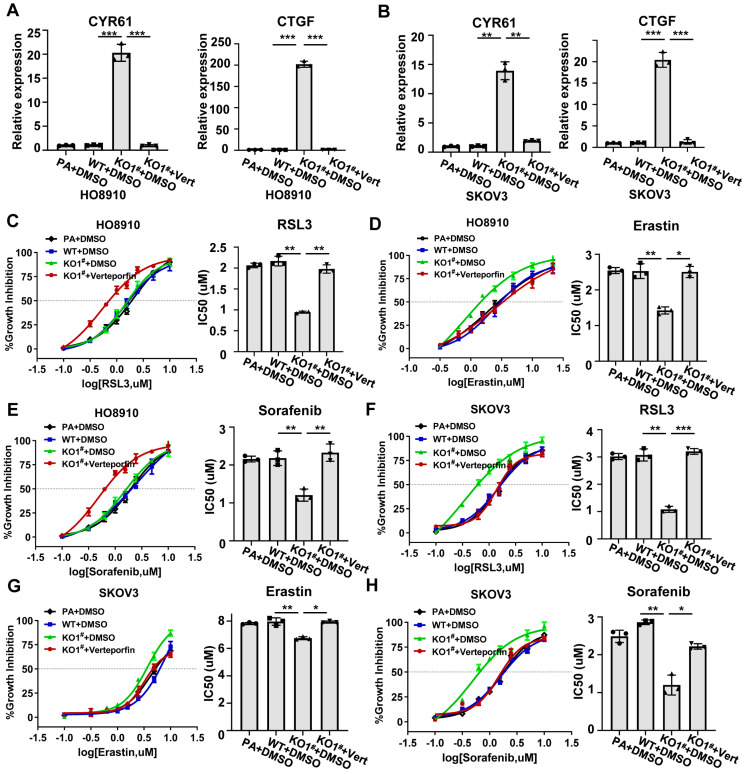
Pharmacological YAP inhibition reverses ferroptosis sensitivity. (**A**,**B**) qRT-PCR assay of CYR61 and CTGF expression post-verteporfin treatment in MED12-KO SKOV3 and HO8910 cells. ** *p* < 0.01 and *** *p* < 0.001. (**C**–**H**) Dose–response curves and IC50 for RSL3, Erastin, and Sorafenib in verteporfin-treated MED12-KO SKOV3 and HO8910 cells. * *p* < 0.05, ** *p* < 0.01, and *** *p* < 0.001. A one-way ANOVA was employed to evaluate statistical differences. PA: parental cells; WT: wild-type; KO: knockout. Error bars represent SD (*n* = 3).

Emerging evidence has established the Hippo–YAP signaling as a key determinant in dictating cellular vulnerability to ferroptotic death in various cancer types [11,20,21]. Building upon our previous findings of MED12’s influence on YAP signaling activation, we hypothesized that MED12 modulates ferroptosis sensitivity through this pathway in ovarian cancer cells. To test this hypothesis, we employed a pharmacological inhibition approach using verteporfin, a well-characterized YAP-TEAD interaction inhibitor, in MED12-knockout (MED12-KO) SKOV3 and HO8910 cells. qRT-PCR and Western blot validated that verteporfin treatment significantly decreased the expression of CYR61 and CTGF in MED12-KO ovarian cancer cells (Figure 4A,B and Figure S6A,B). Treatment with verteporfin significantly attenuated the enhanced ferroptosis sensitivity observed in MED12-KO cells. In HO8910, RSL3 IC50 increased from 0.96 μM to 2.7 μM in verteporfin-treated MED12-KO cells (*p* < 0.01), and Erastin IC50 increased from 2.6 μM to 5.6 μM (*p* < 0.01) in verteporfin-treated MED12-KO cells (Figure 4C–E and Table S1). In SKOV3, RSL3 IC50 increased from 0.77 μM to 3.3 μM in verteporfin-treated MED12-KO cells (*p* < 0.01), and Erastin IC50 increased from 6.2 μM to 7.1 μM (*p* < 0.05) in verteporfin-treated MED12-KO cells (Figure 4F–H and Table S1). These results demonstrate that pharmacological YAP inhibition can reverse MED12-loss-associated ferroptosis vulnerability.

### 2.5. Knockout of MED12 Upregulates the Chromatin Accessibility of YAP–TEAD1 Target Genes Through Enhancing TEAD1 Expression

**Figure 5:**
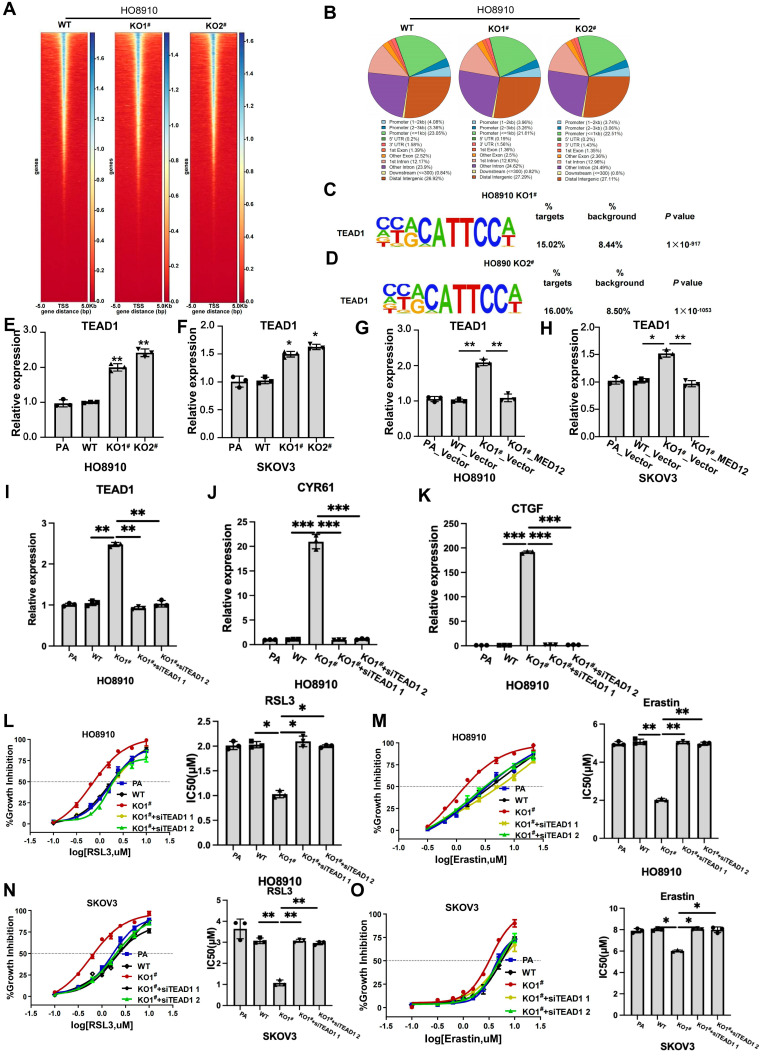
MED12 knockout increases chromatin accessibility at YAP–TEAD1 targets. (**A**) ATAC-seq peak distribution across genomic TSS regions. (**B**) Pie charts showing the genomic distribution of ATAC-seq peak. (**C**,**D**) Motif enrichment analysis showing TEAD1 consensus sequences enriched in MED12-KO cells vs. WT cells. (**E**,**F**) qRT-PCR assay of TEAD1 expression in MED12-KO and WT cells. * *p* < 0.05 and ** *p* < 0.01. (**G**,**H**) qRT-PCR assay of TEAD1 expression in MED12-KO and MED12-reconstituted cells. * *p* < 0.05 and ** *p* < 0.01. (**I**–**K**) qRT-PCR assay of TEAD1, CYR61, and CTGF expression in MED12-KO and MED12-KO + siTEAD1 HO8910 cells. ** *p* < 0.01, and *** *p* < 0.001. (**L**–**O**) Dose–response curves and IC50 for RSL3 and Erastin in MED12-KO and MED12-KO + siTEAD1 SKOV3 and HO8910 cells. * *p* < 0.05 and ** *p* < 0.01. One-way ANOVA test was used to determine statistical significance. PA: parental cells; WT: wild-type; KO: knockout. Error bars represent SD (*n* = 3).

**Figure 6:**
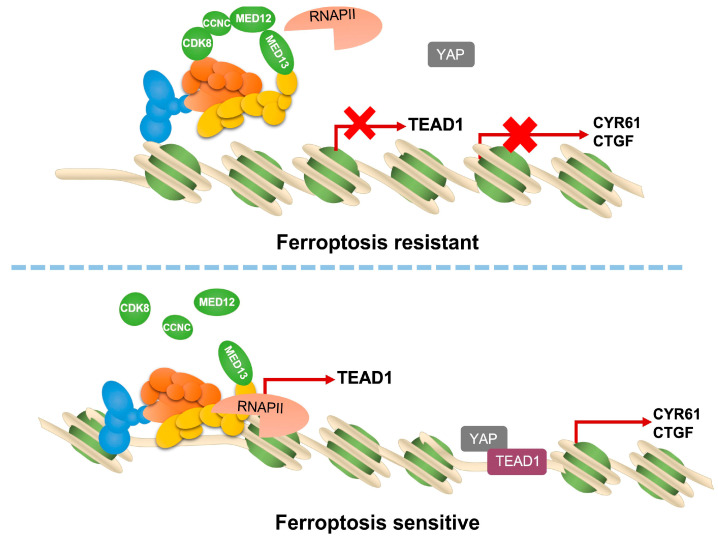
A schematic diagram showing the pathway described in this study.

Previous studies have established that MED12, as a core component of the Mediator complex, exerts its regulatory functions primarily through interaction with CDK8 to modulate transcriptional initiation and elongation. A critical mechanism of the Mediator complex function involves remodeling chromatin accessibility to facilitate transcription factor binding and subsequent gene expression regulation. To investigate whether MED12 influences chromatin architecture in ovarian cancer cells, we conducted Transposase-Accessible Chromatin using sequencing (ATAC-seq) on HO8910 WT and MED12-KO cells (Figure 5A,B). Approximately 30% of identified open chromatin regions localized to promoter regions in each group (±3 kb from transcription start sites) (Figure 5B and Tables S5–S7). De novo motif analysis identified significant enrichment of TEAD1 binding motifs in MED12-KO1 vs. WT HO8910 (15.02% vs. 8.44%, *p* value = 1 × 10^−917^) and in MED12-KO2 vs. WT HO8910 (16.00% vs. 8.50%, *p* value = 1 × 10^−1053^), respectively (Figure 5C,D). These results demonstrate that MED12 deletion creates permissive chromatin states specifically at YAP/TEAD target loci. Building upon our previous RNA-seq data—demonstrating significant upregulation of TEAD1 expression in MED12-knockout ovarian cancer cells compared to MED12 WT ovarian cancer cells (Figure 3A,B), which was subsequently validated by qRT-PCR (Figure 5E,F and Figure S7A,B) and reversed upon MED12 re-expression (Figure 5G,H and Figure S7C,D)—we hypothesized that MED12 modulates YAP–TEAD1 target genes transcriptional activity through TEAD1 regulation. To test this mechanistic model, we performed TEAD1 knockdown using siRNAs in MED12-KO HO8910 and SKOV3 cells. TEAD1 knockdown significantly reduced expression of canonical YAP targets CYR61 and CTGF (Figure 5I–K and Figure S7E–H). Ferroptosis sensitivity assays revealed that TEAD1 knockdown decreased the sensitivity of MED12-KO HO8910 and SKOV3 cells to RSL3 and Erastin (Figure 5N,O). Taken together, these results suggest that TEAD1 is necessary for MED12-KO-induced YAP signaling pathway activation and increased ferroptosis sensitivity (Figure 6).

## 3. Discussion

Our study systematically elucidates the pivotal role of MED12 in regulating ferroptosis sensitivity in epithelial ovarian cancer through the Hippo–YAP signaling pathway. The key findings demonstrate that MED12 knockout significantly enhances ferroptosis sensitivity in EOC cells, which is reversible upon MED12 re-expression. Transcriptomic and chromatin accessibility analyses reveal MED12’s regulation of the Hippo–YAP pathway, particularly through TEAD1-mediated transcriptional activation; moreover, the pharmacological inhibition of YAP or genetic knockdown of TEAD1 attenuates the ferroptosis sensitivity induced by MED12 loss. Collectively, these results establish MED12 as a critical modulator of ferroptosis in EOC.

Our published research suggests that the loss of MED12 induces chemoresistance in EOC cells [19]. While MED12 somatic mutations are relatively infrequent in EOC, TCGA data suggested that the expression of MED12 was lower in ovarian cancers than normal samples and low MED12 expression is associated with poorer disease-free survival. The enhanced ferroptosis sensitivity observed in MED12-deficient EOC cells aligns with emerging evidence that chemotherapy-resistant malignancies often develop metabolic vulnerabilities to ferroptosis [8,9]. Our findings extend this paradigm by identifying MED12 as a central regulator of this process. The mechanistic link between MED12 loss and ferroptosis susceptibility likely stems from the dysregulation of iron homeostasis and lipid peroxidation pathways. This is particularly relevant in ovarian cancer, where platinum resistance frequently coincides with iron accumulation and redox imbalance. Our data suggest that MED12-deficient cells may compensate for chemoresistance by rewiring their metabolic dependencies, inadvertently rendering them susceptible to ferroptosis induction.

The discovery that MED12 modulates the Hippo–YAP pathway provides a novel mechanistic framework for understanding its role in EOC progression. While MED12’s involvement in transcriptional regulation via the Mediator complex is well-documented [12,13], its specific impact on YAP signaling has not been previously characterized. Our ATAC-seq and RNA-seq data demonstrate that MED12 loss increases chromatin accessibility at TEAD1-binding sites, enabling YAP-driven transcription of pro-ferroptotic genes like CYR61 and CTGF (Figure 3 and Figure 5). This remodeling may explain the paradoxical association between chemoresistance and ferroptosis sensitivity, as YAP activation has been implicated in both processes [20,21,22,23,24,25,26]. The consistency of these results across multiple cell lines and clinical specimens underscores the robustness of this mechanism.

Collectively, these results carry substantial therapeutic relevance. The ability of ferroptosis inducers Erastin and RSL3 to target MED12-KO cells (Figure 1) suggests a potential strategy for targeting MED12-deficient EOC. This is particularly compelling given the poor prognosis of chemotherapy-resistant ovarian cancer and the limited treatment options available [2,4,27]. Future studies could explore combinatorial approaches, such as co-using ferroptosis inducers and chemotherapeutic drugs, to exploit the synthetic lethality induced by MED12 loss.

Several unanswered questions warrant further investigation. First, the precise mechanism by which MED12 regulates TEAD1 expression remains unclear. While our data suggest transcriptional control, post-translational modifications or protein–protein interactions may also play a role. Second, the clinical relevance of MED12 expression levels in predicting ferroptosis inducer responses needs validation in larger patient cohorts. Finally, the potential crosstalk between MED12-YAP signaling and other cell death pathways, such as apoptosis or necroptosis, could provide additional therapeutic insights.

In summary, our study uncovers a previously unrecognized role for MED12 in governing ferroptosis sensitivity through the YAP–TEAD1 axis in EOC. These findings not only advance our understanding of the molecular basis of chemoresistance but also offer a promising therapeutic avenue for treating MED12-deficient ovarian cancers. By integrating transcriptional and functional analyses, we provide a comprehensive model that bridges the gap between MED12’s role in transcriptional regulation and its impact on cell fate decisions. This work lays the foundation for future research aimed at exploiting ferroptosis induction as a strategy to overcome chemotherapy resistance in ovarian cancer.

## 4. Materials and Methods

### 4.1. Cell Culture and Reagents

The human EOC cell lines SKOV3 and HO8910 were acquired from the Sun Yat-sen University Cancer Center. Incubation was carried out at 37 °C with 5% CO_2_ and humidity, while cell growth relied on DMEM fortified with 10% fetal bovine serum (FBS) and 1% penicillin/streptomycin. The compounds used in this study, including ferroptosis inducers (RSL3, Erastin), inhibitors (Ferrostatin-1, Z-VAD-FMK, Necrosulfonamide), and Verteporfin, were sourced from MedChemExpress (Shanghai, China).

### 4.2. Generation of MED12-Knockout Cell Lines

For MED12 knockout, two guide RNAs were designed: gRNA#1 (AGGATTGAAGCTGACGTTCT) and gRNA#2 (GATTGCTGCATAGTAGGCAC). SKOV3 and HO8910 cells, seeded in six-well plates and grown to 70–80% confluence, were co-transfected per well with 1 mg of each MED12 sgRNA plasmid, 1 mg of the pSpCas9(BB)-2A-GFP plasmid, and 5 mL Lipofectamine2000 (Thermo Fisher Scientific, Waltham, MA, USA). Using GFP fluorescence as a marker, transfected cells were isolated via FACS analysis two days after the transfection step and dispensed into 96-well plates. Single-cell-derived clones were expanded after validation of MED12 knockout via Western blot and Sanger sequencing, establishing the stable KO lines.

### 4.3. Cell Viability and Ferroptosis Assays

To assess survival, we seeded 3000 cells into each well of a 96-well plate, exposed to a dose range of ferroptosis inducers (0.1–10 μM) for 48 h, and then assessed using the Cell Counting Kit-8 (Dojindo, Guangzhou, China) as per the manufacturer’s instructions. We calculated the half-maximal inhibitory concentration (IC50) using non-linear regression models within the GraphPad Prism 9.0 software. In separate inhibitor experiments, cells were co-treated with Ferrostatin-1 (5 μM), Z-VAD-FMK (20 μM), or Necrosulfonamide (10 μM).

### 4.4. RNA Sequencing and Bioinformatics Analysis

Adhering to the standard product guidelines, RNA harvesting was carried out with the TRIzol reagent system (Invitrogen, Shanghai, China). RNA quantity and purity were measured with a NanoDrop ND-1000 spectrophotometer (Thermo Fisher Scientific, Waltham, MA, USA). Integrity was verified using an Agilent Bioanalyzer 2100 (Agilent, Santa Clara, CA, USA) (RIN > 7.0) and confirmed by denaturing agarose gel electrophoresis. Poly(A)+ RNA was then isolated from 1 μg of total RNA with Dynabeads Oligo(dT)25 (Thermo Fisher Scientific, Waltham, MA, USA) through two rounds of purification. Subsequently, the purified RNA was fragmented for 5–7 min at 94 °C using the Magnesium RNA Fragmentation Module (NEB, Ipswich, MA, USA). First-strand cDNA was synthesized from the cleaved RNA fragments using SuperScript™ II Reverse Transcriptase (Invitrogen, Shanghai, China). Second-strand synthesis was performed with *E. coli* DNA polymerase I, RNase H (NEB, Ipswich, MA, USA), and dUTP (Thermo Fisher Scientific, Waltham, MA, USA) to generate U-labeled DNA. Following end repair and A-tailing, indexed adapters were ligated. Post-ligation size selection was carried out with AMPureXP beads. The U-labeled strand was digested with UDG (NEB, Ipswich, MA, USA), and the adapter-ligated fragments were amplified by PCR under the following conditions: 95 °C for 3 min; 8 cycles of 98 °C for 15 s, 60 °C for 15 s, 72 °C for 30 s; final extension at 72 °C for 5 min. The resulting cDNA libraries had an average insert size of 300 ± 50 bp and were subjected to 2 × 150 bp paired-end sequencing on an Illumina NovaSeq 6000 platform (LC-Bio) (Illumina, San Diego, CA, USA) according to the manufacturer’s instructions. Raw sequencing reads were processed with fastp to trim adapters and remove low-quality or undetermined bases using default parameters, followed by quality verification. Clean reads were then aligned to the Homo sapiens GRCh38 reference genome using HISAT2 (version 2.2.1). Subsequently, transcript assembly for each sample was performed with StringTie under default settings. To build a unified dataset, we combined transcriptomes from every sample using the gffcompare tool (version 2). Following this reconstruction, we applied StringTie to quantify transcript abundance, specifically calculating FPKM values to represent mRNA levels. Finally, we utilized the edgeR package (version 4) to pinpoint differentially expressed genes; candidates were filtered based on a parametric F-test (*p* < 0.05) and a fold-change magnitude greater than two (FC > 2 or <0.5).

### 4.5. ATAC-Seq and Chromatin Analysis

A total of 50,000 cells were processed using the ATAC protocol. Library quantification was performed on a Bioptic Qsep100 Analyzer (Bioptic, La Canada Flintridge, CA, USA) prior to 150 bp paired-end sequencing. Subsequent data processing followed the ENCODE ATAC-seq pipeline for quality control and statistical analysis, which generated aligned reads and enrichment profiles. These reads were mapped to the human reference genome (hg38), and differential chromatin accessibility sites were identified using the DiffBind R package (version 3.21).

### 4.6. Quantitative PCR

Following total RNA isolation using TRIzol reagent (Invitrogen, Shanghai, China), cDNA was synthesized with the PrimeScript RT Reagent Kit (Takara, Beijing, China). Gene-specific primers were designed in Primer Premier 5.0. Quantitative PCR was then carried out with SYBR Green Master mix (Takara, Beijing, China) in accordance with the manufacturer’s instructions.

### 4.7. Statistical Analysis

All experiments included at least three biological replicates, with data expressed as mean ± SD. All computations were executed in GraphPad Prism 9.0, utilizing Student’s *t*-test or ANOVA with Tukey’s post hoc adjustment; values of *p* < 0.05 were deemed significant. To ensure reproducibility, key raw data have been deposited in the Research Data Deposit platform (www.researchdata.org.cn; approval number RDDB2026381477).

## Data Availability

The raw data supporting the conclusions of this article will be made. available by the authors on request. RNA-seq and ATAC-seq data are available publicly at http://www.ncbi.nlm.nih.gov/geo (GEO accession numbers: GSE315124 and GSE315235) (accessed on 1 January 2026).

## Supplementary Materials

The following supporting information can be downloaded at: https://www.mdpi.com/article/10.3390/ijms27021020/s1.

## Abbreviations

The following abbreviations are used in this manuscript:

EOC	epithelial ovarian cancer
MED12	Mediator complex subunit 12
EMT	epithelial–mesenchymal transition
ABC	ATP-binding cassette
KO	knockout
WT	wild-type
GSEA	Gene Set Enrichment Analysis
ATAC-seq	assay for Transposase-Accessible Chromatin using sequencing
SD	standard deviation
