# Supplementary material for: MED12 Dictates Epithelial Ovarian Cancer Cell Ferroptosis Sensitivity via YAP–TEAD1 Signaling
Source: Int J Mol Sci. 2026 Jan 20;27(2):1020. doi: 10.3390/ijms27021020 (PMC12842402; doi:10.3390/ijms27021020)
Supplement: Supplementary file 1 [file ijms-27-01020-s001.zip › Supplemental Figures.pdf]

Supplemental Figures

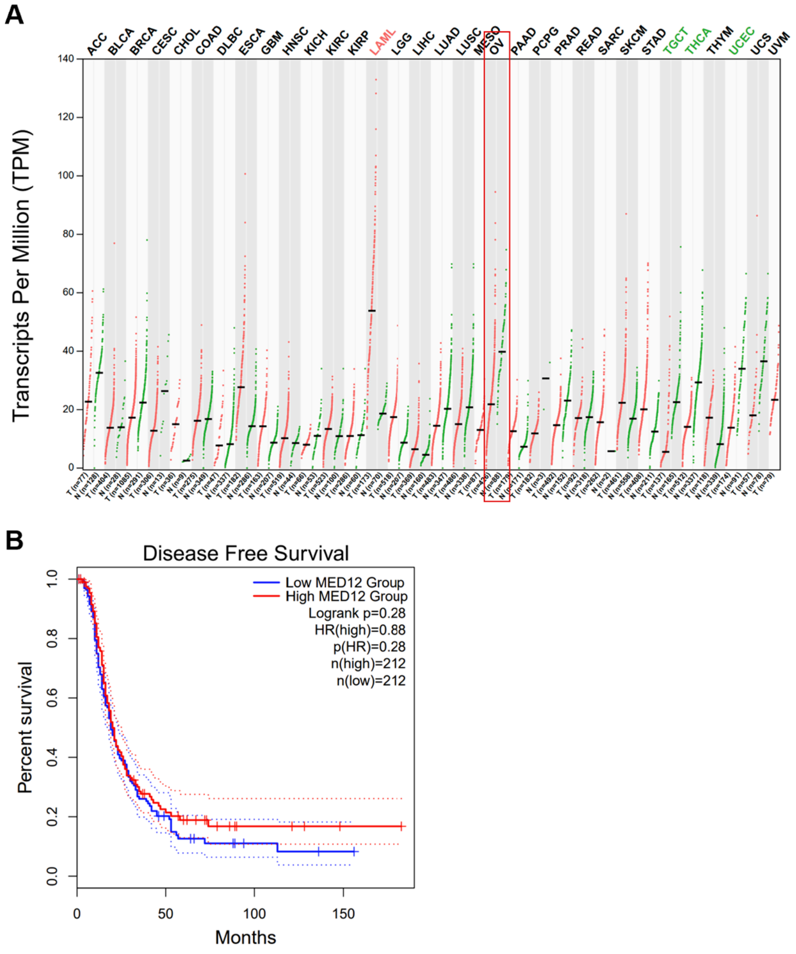

Figure S1. TCGA analysis of MED12 in ovarian cancers. (A) The gene expression profile of MED12 across all tumor samples and paired normal tissues. Each dot represents expression of samples. (B) Disease Free Survival analysis of MED12 in ovarian cancers.

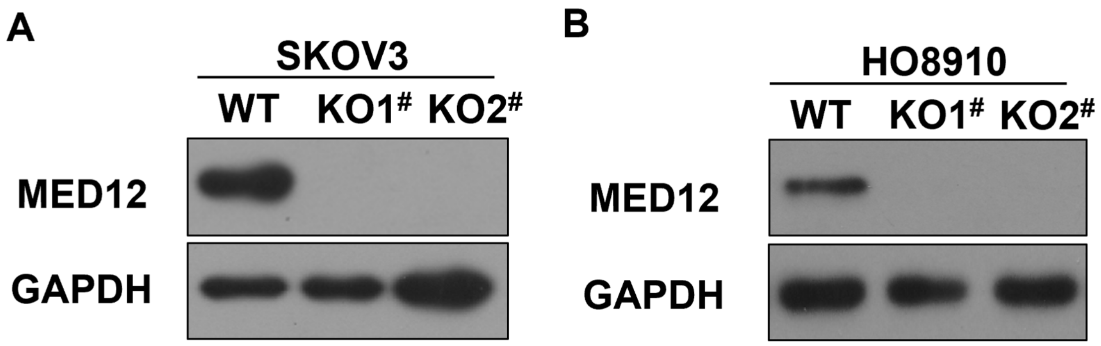

Figure S2. Western blot analysis of MED12 expression in SKOV3 and HO8910 MED12 knockout cells.

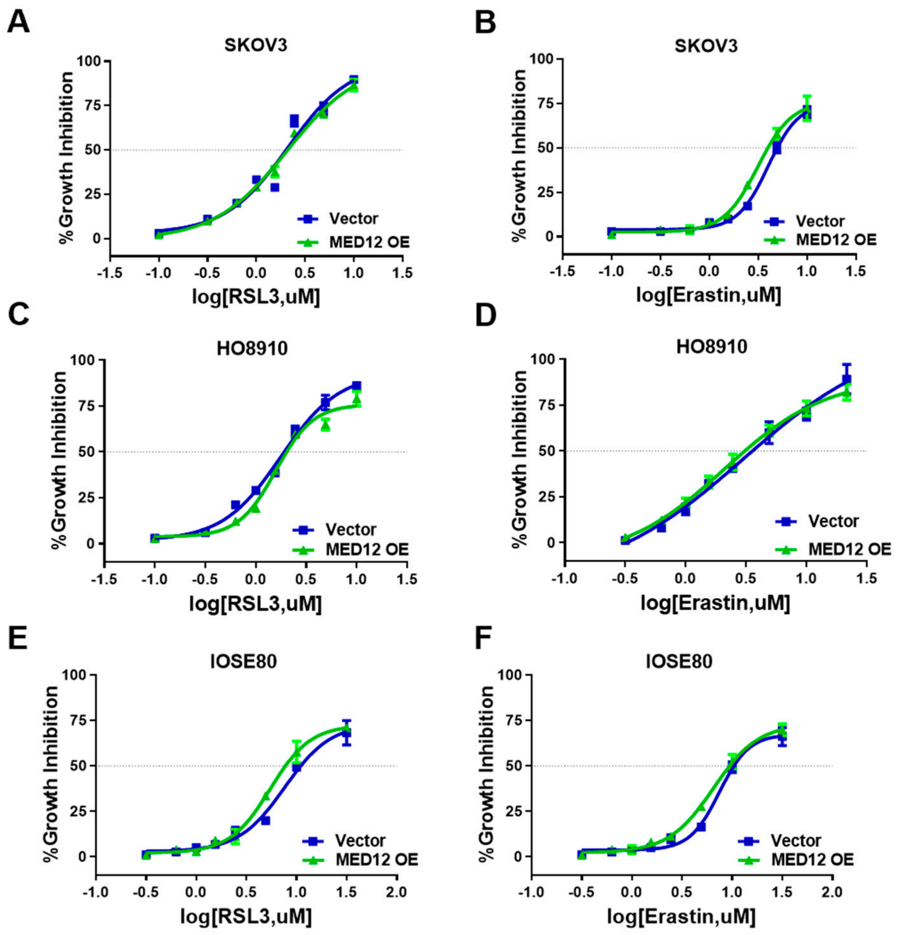

Figure S3. Dose-response curves for RSL3 and Erastin treatment in Vector and MED12 OE cells. (A-D) Dose-response curves for RSL3 and Erastin treatment in Vector and MED12 OE SKOV3 and HO8910 cells. (E, F) Dose-response curves for RSL3 and Erastin treatment in Vector and MED12 OE IOSE80 cells. OE: overexpression.

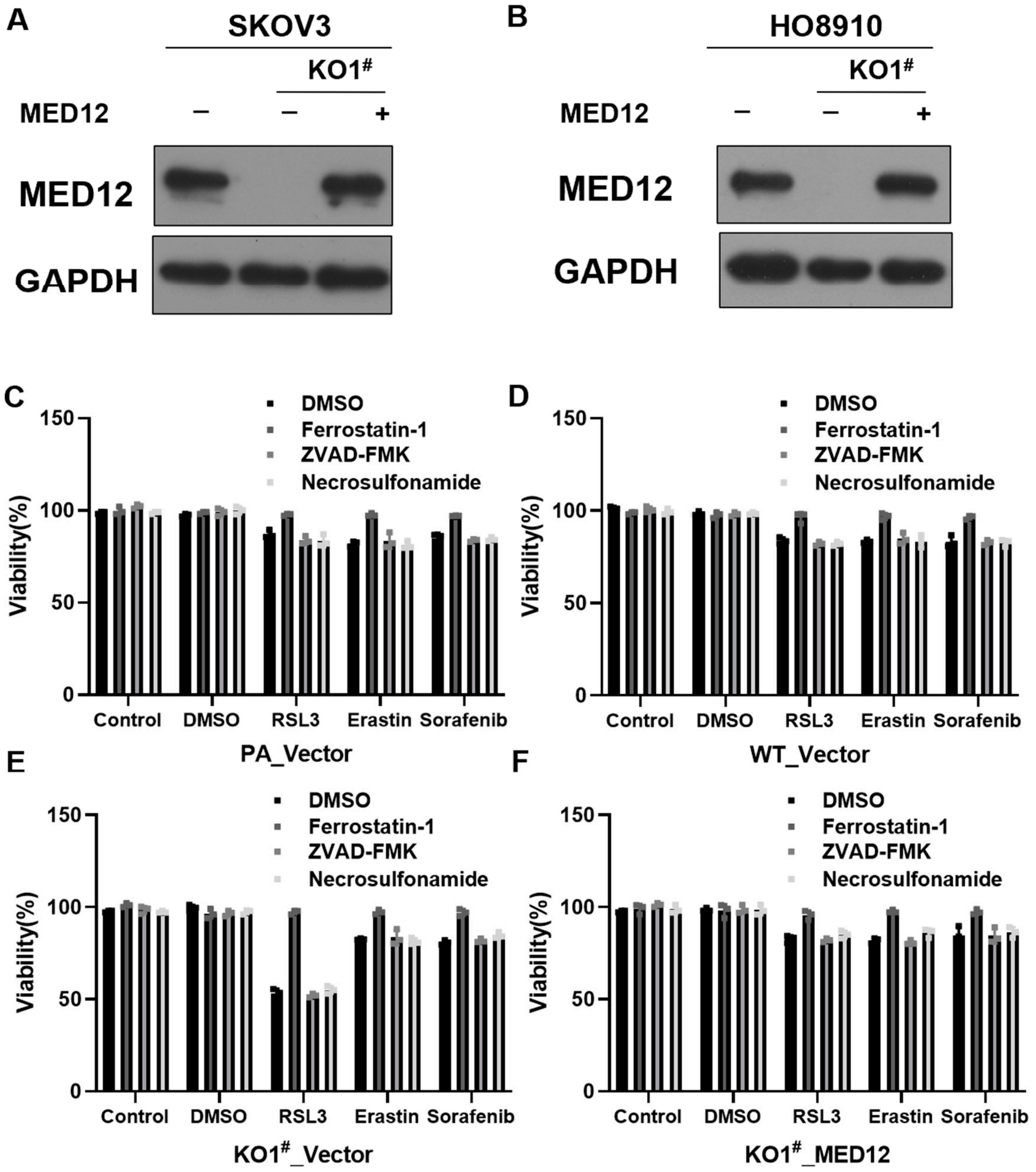

Figure S4. MED12 re-expression rescues ferroptosis sensitivity. (A, B) Western blot analysis of MED12 expression in SKOV3 and HO8910 MED12 knockout and rescue cells. (C-F) Cell death inhibition assays showing ferroptosis specificity in HO8910. Error bars rep-resent SD (n=3).

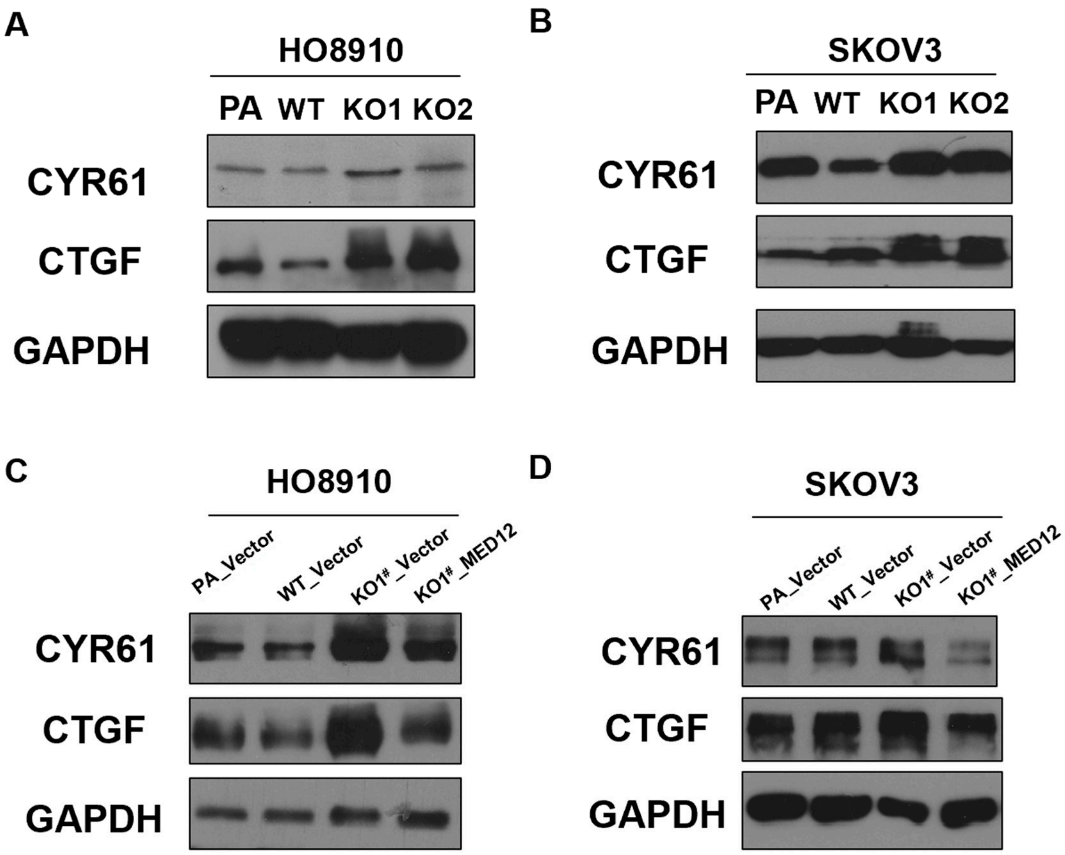

Figure S5. Western blot reveals MED12 regulation of Hippo-YAP signaling. (A, B) Western blot assay of CYR61 and CTGF expression in MED12-KO HO8910 and SKOV3 cells. (C, D) Western blot assay of CYR61 and CTGF expression in MED12-KO and MED12-reconstituted HO8910 and SKOV3 cells.

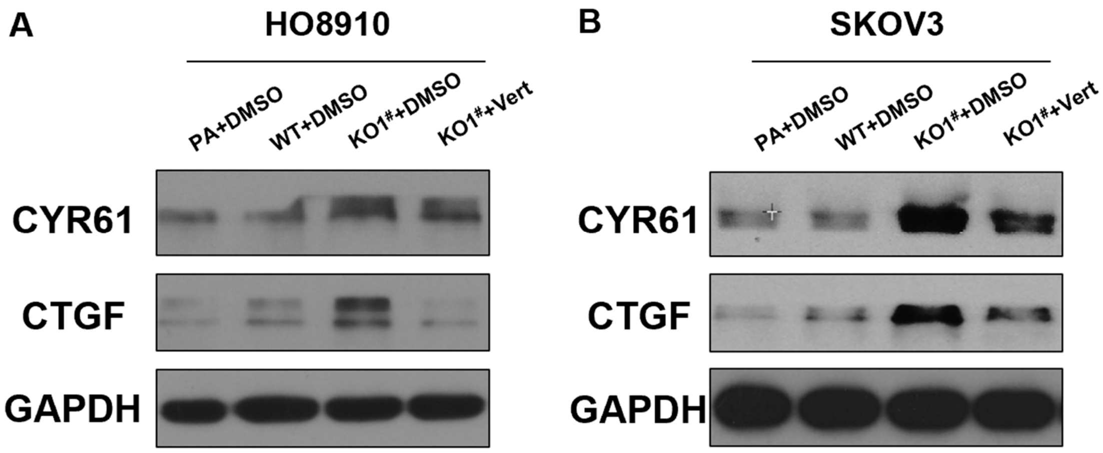

Figure S6. Western blot assay of CYR61 and CTGF expression post-verteporfin treatment in MED12-KO SKOV3 and HO8910 cells.

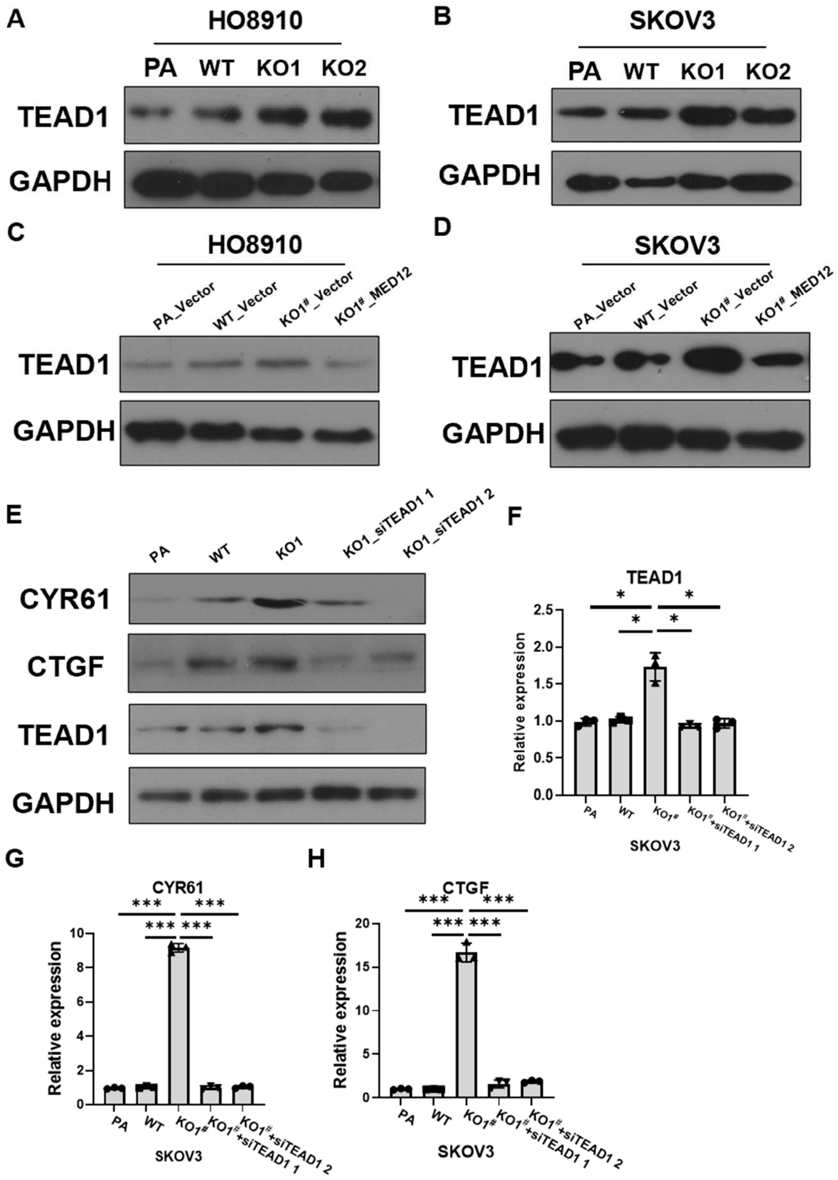

Figure S7. MED12 knockout increases chromatin accessibility at YAP-TEAD1 targets. (A, B) Western blot assay of TEAD1 expression in MED12-KO and WT cells. (C, D) Western blot assay of TEAD1 expression in MED12-KO and MED12-reconstituted cells. (E) Western blot assay of TEAD1, CYR61 and CTGF expression in MED12-KO and MED12-KO+siTEAD1 SKOV3 cells. (F-H) qRT-PCR assay of TEAD1, CYR61 and CTGF expression in MED12-KO and MED12-KO+siTEAD1 SKOV3 cells. Error bars represent SD (n=3). *P<0.05 and ***P<0.001.
